# High Positivity Rate for *Leptospira* Infection in Symptomatic Urban Owned Dogs in Guayaquil, Ecuador

**DOI:** 10.3390/tropicalmed11060145

**Published:** 2026-05-26

**Authors:** Solon Alberto Orlando, Naomi Mora Jaramillo, Ariana Montenegro Pesántez, Melissa Joseth Carvajal-Capa, Jose Julián Zuñiga-Velarde, Silvia Tafur, Miguel Angel Garcia-Bereguiain

**Affiliations:** 1 Instituto Nacional de Salud Pública e Investigación, Guayaquil, Ecuador; 2 Universidad Ecotec, Km 13.5 Samborondón, Samborondón 092302, Ecuador; 3 Universidade Lusofona, Lisboa, Portugal; 4 Universidad Católica Santiago de Guayaquil, Guayaquil, Ecuador; 5 Clinica Veterinaria Dr Pet, Guayaquil, Ecuador; 6 Tafur Animal Care, Guayaquil, Ecuador; 7 One Health Research Group, Universidad de Las Américas, Quito 170516, Ecuador

**Keywords:** leptospirosis, qPCR, dogs, Ecuador, One Health

## Abstract

Leptospirosis is a zoonotic disease caused by spirochete bacteria of the genus *Leptospira*, with a wide global distribution. In Ecuador, leptospirosis is endemic, particularly in low-resource tropical areas, and multiple animal reservoirs have been identified either in rural or urban areas, including stray dogs. In this study, a total of 81 domestic dogs presenting clinical manifestations compatible with leptospirosis were recruited at the Municipal Center for Animal Welfare in Guayaquil, Ecuador, in 2023. A survey regarding clinical, demographic, and environmental risk factors was filled in by every dog’s owner; urine and blood samples were collected for pathogenic *Leptospira* diagnosis by qPCR for *lipL32*, *rrs*, and *secY* gene targets. A very high (62.96%) positivity rate for *Leptospira* infection was found. Almost 90% of the dogs were not vaccinated against *Leptospira*. Although the animals exhibited multiple clinical signs, none showed a statistically significant association with *Leptospira* positivity, confirming the nonspecific presentation of the disease and its potential for misdiagnosis. The consumption of bulk food emerged as a significant environmental risk factor only in the multivariate logistic regression and not in the univariate analysis, suggesting the need for improved food safety practices. Moreover, we reported very frequent close-contact behaviors between owners and dogs. Overall, our study underscores the potential role of owned urban dogs as reservoirs of *Leptospira* in the city of Guayaquil in Ecuador, emphasizing the need for public health policies to increase awareness and improve diagnosis in domestic animals under a comprehensive One Health vision.

## 1. Introduction

Leptospirosis is a zoonotic disease caused by spirochete bacteria of the genus *Leptospira*, and it is present in all continents with a wide global distribution [1]. In Ecuador, the disease is endemic, particularly in low-resource tropical settings of the central–northern coastal region and also during the rainy season in the southern Amazon region [2,3]. In Guayaquil, antibodies against *Leptospira* have been detected in 94.7%, 82.1%, and 83.6% of stray dogs, cats, and rats, indicating active synantropic reservoirs [4].

Some animals can chronically carry *Leptospira* spp. asymptomatically. Its epidemiological importance lies in the fact that humans can become infected through direct contact with the urine of infected animals or indirectly by exposure to contaminated environments, such as freshwater or soil made moist with urine [5]. Moreover, a recent study conducted in Ecuador showed an epidemiological link to infected dogs in a human leptospirosis outbreak [6].

The most common clinical signs of leptospirosis in dogs include lethargy, anorexia, vomiting, diarrhea, and abdominal pain. Leptospirosis may also present with oliguria, dyspnea or tachypnea, reluctance to move, respiratory difficulty, jaundice, pale mucous membranes, fever or hypothermia, peripheral lymphadenopathy, polydipsia and polyuria, stiff gait, adipsia, weight loss, and brownish-red urine [7]. Dogs with renal or hepatic disease may present clinical signs like those caused by leptospirosis, with the most frequent being anorexia, vomiting, lethargy, jaundice, and diarrhea [8]. Hyperbilirubinemia and pulmonary hemorrhagic syndrome are associated with a poor prognosis, with the latter having a reported mortality rate of up to 70% [9]. It has been shown that dogs positive for *Leptospira* have shorter survival times compared to healthy dogs, highlighting the severity of this infection [10].

Multiple studies have explored risk factors for leptospirosis in dogs, including age, breed, sex, vaccination status, geographic location, indoor and outdoor ownership, and exposure to other dogs, rats, or flooding [8,11,12]. The success of early antimicrobial treatment and supportive care for leptospirosis recovery in dogs depends on timely and accurate diagnosis, which can be challenging and is based on multiple criteria. For fast and accurate diagnosis, qPCR has been recommended [13,14].

Although a high prevalence of leptospirosis has already been reported in free-roaming dogs in Guayaquil city, no studies have focused on owned dogs, even though leptospirosis is endemic in this area, and vaccination against *Leptospira* is very unusual. Against this backdrop, the current study aimed to examine *Leptospira* infection in symptomatic owned dogs from Guayaquil city, as well as its clinical signs and associated risk factors.

## 2. Materials and Methods

### 2.1. Study Design and Population

A cross-sectional observational study enrolling 81 dogs was conducted between October 2022 and January 2023 at the Municipal Center for Animal Welfare in Guayaquil, Ecuador, to estimate the *Leptospira* spp. positivity rate in owned dogs with clinical manifestations compatible with leptospirosis and analyze associated clinical and epidemiological factors coming from neighborhoods all over the city. The inclusion criteria included the presence of two or more of the following clinical signs compatible with leptospirosis: fever, diarrhea, vomiting, turbid urine, jaundice, or hemorrhagic manifestations. Those clinical signs have been described as the most common ones associated with leptospirosis [9,15,16]. Dogs were excluded if complete paired urine and blood samples could not be obtained or if clinical histories were incomplete.

### 2.2. Data and Sample Collection

Data on demographic characteristics (age, sex), vaccination status, clinical signs, susceptibility factors, and the level of dog–owner contact were collected through a combination of owner interviews and clinical records.

Whole blood samples with anticoagulant were collected via venipuncture of the cephalic, saphenous, or jugular veins. Urine samples were obtained aseptically by ultrasound-guided cystocentesis.

### 2.3. DNA Extraction and Real-Time PCR (qPCR) for Pathogenic Leptospira Detection

DNA was extracted from all urine and whole blood samples using the PureLink Genomic DNA Mini Kit (Invitrogen, Waltman, MA, USA), according to the manufacturer’s instructions. Pathogenic *Leptospira* spp. detection was performed using a multiplex real-time PCR (qPCR) assay as previously described [17]. Briefly, the assay simultaneously targeted the *lipL32*, *secY*, and *rrs* (16S rRNA) genes. A *β-actin* gene was included as an internal control to monitor for PCR inhibition. The qPCR reaction mixture and cycling conditions were identical to those in the referenced protocol. Each run included positive controls and non-template controls. A sample was considered positive for *Leptospira* if it produced a cycle threshold (Ct) value of ≤40 for any of the three target genes (*lipL32*, *secY*, or *rrs*).

### 2.4. Data Analysis

All data were organized in Microsoft Excel and analyzed using IBM SPSS Statistics 26. Descriptive statistics were used to summarize the characteristics of the study population and to estimate the positivity rate for *Leptospira* spp. A Pearson’s Chi-Square test was used to evaluate bivariate associations between categorical variables (e.g., age, sex, vaccination status, individual clinical signs, risk factors) and qPCR positivity.

Variables with a *p*-value < 0.1 in the bivariate analysis were included in a multivariate binary logistic regression model to identify independent factors associated with *Leptospira* spp. positivity, with results expressed as adjusted odds ratios (aORs) with 95% confidence intervals (CIs). A *p*-value of <0.05 was considered statistically significant.

Finally, a descriptive frequency analysis was performed to characterize the nature and extent of contact between owners and their dogs that tested positive for *Leptospira* spp.

## 3. Results

### 3.1. Leptospira spp. Diagnosis by qPCR

During the study period, 81 dogs meeting the inclusion criteria were enrolled. Of these, 51 tested positive for one or more *Leptospira* spp. genes, as assessed by qPCR, yielding a positivity rate of 62.96% (51/81). One sample was positive for the *lipL32* gene in urine, while no blood samples tested positive for *lipL32*. Additionally, 15 urine samples and 14 blood samples were positive for the *rrs* gene. The *SecY* gene was detected in 20 urine samples and 26 blood samples. Information regarding positivity for each type of sample, each gene, and gene combinations is detailed in Table 1, Table 2 and Table 3 (access to the raw data is available upon request to the authors).

### 3.2. Demographic and Vaccination Status

The associations between dog demographics and *Leptospira* spp. positivity are shown in Table 4. For the overall dog population included in this study, 87.6% were unvaccinated. Moreover, among dogs positive for leptospirosis, 13.7% (7/51) had been vaccinated against the *L. icterohaemorrhagiae* and *L. canicola* serovars. No significant association was found between sex and *Leptospira* spp. infection, with values of 63.2% (24/38) and 62.8% (27/43) for males and females, respectively. Regarding age, a statistically significant association was found between the age of the dogs and *Leptospira* spp. positivity, with a *p*-value < 0.05 (Pearson Chi-Square analysis, Table 4). Among dogs ≥ 1 year, 53.7% (29/54) tested positive, while among dogs < 1 year, 81.5% (22/27) tested positive.

### 3.3. Clinical Manifestations in Dogs Found Positive for Leptospira spp. by qPCR

**Figure 1:**
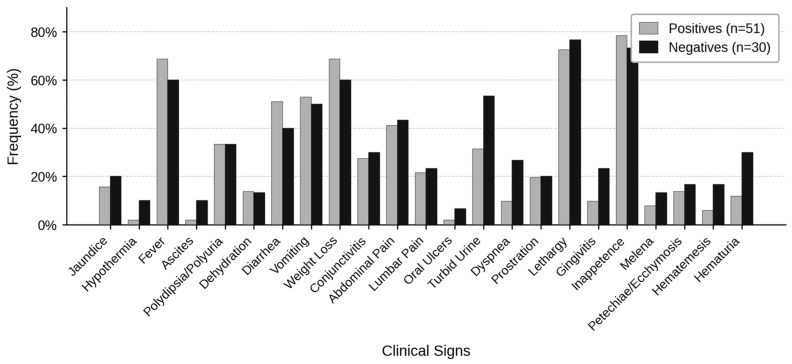
Frequency distribution of clinical signs in dogs positive and negative for leptospirosis by qPCR included in this study.

The frequency of clinical signs observed in positive dogs is summarized in Figure 1. The most common systemic signs were anorexia (78.4%) and lethargy (74.5%), followed by weight loss and fever (68.6% each), indicative of systemic illness. Gastrointestinal signs such as vomiting (52.9%) and diarrhea (51.0%) were also highly prevalent. Signs suggestive of renal involvement, such as turbid urine (31.4%) and polyuria/polydipsia (33.3%), were also observed. Hemorrhagic manifestations were present in 33.3% of cases, while icterus was less common (15.7%). Rare manifestations, each observed in fewer than 10% of the dogs, encompassed gingivitis, dyspnea, constipation, oral ulcers, hypothermia, and ascites.

### 3.4. Environmental Risk Factor Analysis

**Figure 2:**
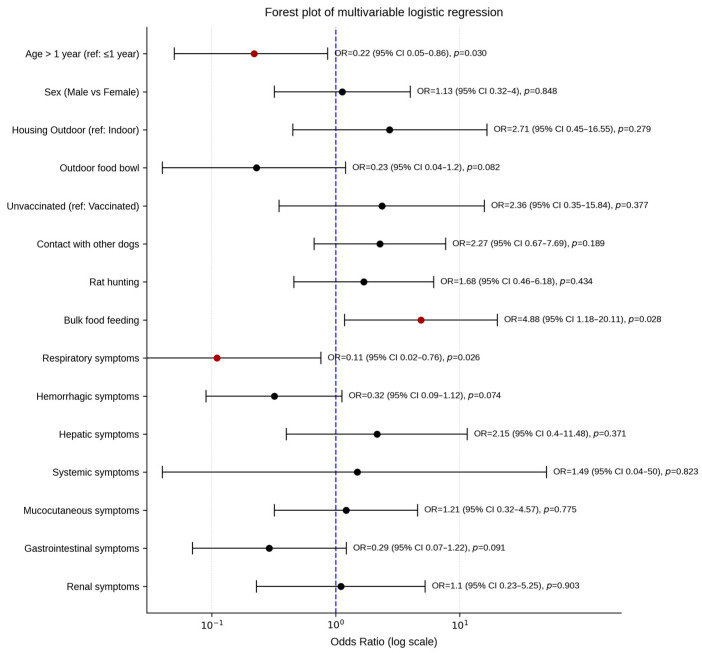
Forest plot of the multivariable binary logistic regression for factors associated with *Leptospira* spp. infection in the dogs included in this study. Forest plot showing the odds ratios (ORs) and 95% confidence intervals (CIs) for clinical signs associated with PCR-confirmed *Leptospira* spp. infection in dogs. Red dots indicate statistically significant associations (*p* < 0.05); the vertical blue line represents the null value (OR = 1.0).

Univariate analysis of environmental and husbandry risk factors did not reveal any statistically significant associations with *Leptospira* spp. positivity (Table 5). Additionally, a multivariate binary logistic regression model was constructed to identify independent predictors of *Leptospira* spp. positivity (Figure 2). After adjusting for other variables, dogs over one year of age had significantly lower odds of infection (aOR = 0.22, 95% CI: 0.05–0.86, *p* = 0.030). The presence of respiratory symptoms was also negatively associated with positivity (aOR = 0.11, 95% CI: 0.02–0.76, *p* = 0.026). Conversely, consumption of bulk food (meaning food sold in open sacks or large containers) was independently associated with a significantly higher odds of infection in the multivariate binary logistic regression analysis (aOR = 4.88, 95% CI: 1.18–20.11, *p* = 0.028).

### 3.5. Owner Exposure and Contact Behaviors with Dogs Included in the Study

The frequency of close-contact behaviors between owners and their *Leptospira*-positive dogs is detailed in Table 6. The most common behavior was owners kissing their dog on the face (76.5%), followed by the dog licking the owner’s face (68.6%). Half of the owners (51.0%) reported kissing their dog on the mouth.

## 4. Discussion

This study identified a high positivity rate for *Leptospira* infection in both urine and blood from symptomatic owned dogs in Guayaquil city by using real-time PCR targeting the *lipL32*, *rrs*, and *secY* genes for pathogenic *Leptospira*. Interestingly, the presence of infection in blood, urine, or both, depending on the dogs, revealed different stages of leptospirosis. Moreover, bacterial loads were very low in general, as revealed by the high Ct values obtained in the PCR tests. In this sense, the variability observed between the gene markers’ positivity is explained by the intrinsic limitations of reproducibility in PCR for Ct values close to the limit of detection.

These findings are particularly striking when considering that leptospirosis is often under-suspected in clinical practice, confirming its underdiagnosed nature in owned dogs and their potential role as reservoirs of *Leptospira* in an urban setting like Guayaquil. Previous studies have documented that leptospirosis is endemic in Ecuador, particularly in tropical, low-resource settings, with higher prevalence observed in central–northern coastal cantons [2,3,4]. This study was strategically conducted at a municipal hospital in Guayaquil, the most populated city in Ecuador, located in the coastal province of Guayas. Most pet owners visiting this facility come from communities with high poverty rates and limited access to basic services such as clean water and sanitation. These areas also experience a high presence of stray dogs. These conditions, combined with the selection of owned dogs presenting symptoms suggestive of leptospirosis, resulted in the high proportion of positive cases observed.

Interestingly, almost 90% of the dogs included in the study were unvaccinated against leptospirosis, underscoring a combination of lack of knowledge of this zoonotic threat and lack of resources for vaccination among owners in an endemic area. Moreover, seven dogs vaccinated against *L. icterohaemorrhagiae* and *L. canicola* serovars were positive for *Leptospira* spp.; while vaccines containing certain *Leptospira* strains can provide some cross-protection against other strains [18,19] and are effective in reducing hepatitis and kidney failure, they do not guarantee complete protection [20,21]. Unfortunately, the region has a wide diversity of circulating serovars for which commercial vaccines are not available [4].

Dogs younger than one year are more susceptible to infectious diseases because puppies are born with a functional but still immature immune system [22]. The statistically significant association between age and *Leptospira* positivity in this study, with younger dogs showing higher infection rates, supports this vulnerability, reinforcing the importance of early vaccination and protection measures for puppies. Our findings suggest the need to review vaccination schedules and formulations in Ecuador, as reported in studies primarily conducted in European countries [23].

Unlike some studies reporting that male dogs with outdoor access are significantly associated with leptospirosis [24] this study found no statistically significant associations with sex or several environmental risk factors like indoor/outdoor living arrangements, contact with other dogs, or rodent hunting behavior. This lack of association may reflect local epidemiological dynamics or suggest that risk factors act synergistically or vary depending on local contexts [25]. Nevertheless, male behavior such as urine marking and mounting may still contribute to their role as reservoirs and transmitters [17,26]. However, the consumption of bulk food emerged as an independent risk factor significantly associated with higher odds of infection, though this was only in the multivariate logistic regression and not in the univariate analysis. In this sense, food contamination by rodents has been reported as a factor associated with leptospirosis [27,28]. Inadequate food safety practices, such as bulk food sold in open sacks or large containers that remain exposed for extended periods, make it more accessible to rodents and other species, potentially transforming it into a fomite for leptospirosis transmission [29].

Leptospirosis presents a wide range of signs and symptoms that can be mistaken for other diseases [30], and the initial febrile phase is usually nonspecific [9]. In this study, positive dogs exhibited various clinical signs, most commonly fever, anorexia, lethargy, weight loss, vomiting, and diarrhea. Despite this clinical diversity, no individual clinical sign showed a statistically significant association with *Leptospira* positivity, suggesting that the disease can present in a nonspecific manner and may be easily confused with other conditions. However, the presence of respiratory symptoms was negatively associated with leptospirosis, indicating that dogs with leptospirosis are less likely to show respiratory signs. This aligns with studies where respiratory involvement has either not been reported [30] or observed at low incidence [31]. However, when respiratory signs such as pulmonary hemorrhagic syndrome do occur, they are typically associated with high mortality and appear during advanced or severe stages of the disease [9,32].

*Leptospira* can survive in water or moist soil for weeks or even months [33,34], and humans can contract the disease through direct or indirect exposure to contaminated environments or infected animals [35]. In Ecuador, stray dogs have been identified as important reservoirs of *Leptospira* both in rural and urban settings [6]. Moreover, asymptomatic domestic dogs may act as environmental sentinels for leptospirosis surveillance [36]. Our findings also support the role of owned dogs with clinical manifestations compatible with leptospirosis as reservoirs of *Leptospira* in the urban setting of Guayaquil. In this sense, the high frequency of close-contact dog–owner behaviors documented in this study, no matter the poor health conditions of the dogs, suggests that poor hygiene practices combined with close human–dog interaction could represent an important epidemiological risk in urban settings where leptospirosis is endemic, like in Guayaquil city.

In conclusion, this study revealed a high positivity rate for *Leptospira* infection in owned dogs with clinical signs compatible with leptospirosis in Guayaquil, Ecuador. Although the animals exhibited multiple clinical signs, none showed a statistically significant association with *Leptospira* positivity, confirming the syndromic presentation of the disease and its potential for misdiagnosis. The consumption of bulk food was an environmental risk factor for *Leptospira* infection only in the multivariate logistic regression model and not in the univariate analysis, suggesting the need for improved food safety practices. The frequent close-contact behavior between owners and infected dogs underscores the public health importance of leptospirosis in this urban setting and emphasizes the need for increased awareness and improved diagnostic protocols. Overall, leptospirosis is an endemic disease in Guayaquil city, and comprehensive One Health interventions, including public health policies to increase awareness of the risk among dog owners and identification of domestic animals as potential reservoirs, are needed to reduce transmission risk to humans.

## Figures and Tables

**Table 1:** Distribution of *Leptospira* PCR-positive animals by sample type.

Classification	n	%
Positive in urine only	14	27.5
Positive in blood only	19	37.3
Positive in urine and blood	18	35.3

**Table 2:** Distribution of *Leptospira* PCR-positive animals for each gene target.

Gene Target	n	%
*rrs* (*16S*)	25	49.0
*secY*	39	76.5
*LipL32*	1	2.0

**Table 3:** Distribution of *Leptospira* PCR-positive animals for each gene target combination.

Combination	n	%
*rrs* + *secY*	13	25.5
*rrs* + *secY* + *LipL32*	1	2.0

**Table 4:** Pearson Chi-Square analysis of dog demographics associated with leptospirosis.

Factor	Category	Pearson Chi-Square	*p*-Value	OR (95% CI)
Sex	Male/Female	0.001	1.00	1.01 (0.41–2.51)
Vaccination	Yes/No	0.24	0.62	1.43 (0.34–6.01)
Age Group *	<1 year/≥1 year	5.96	0.01 *	3.79 (1.25–11.49)

Note: * significant at *p*-value < 0.05; OR is odds ratio value; 95% CI is 95% confidence interval of lower limit and upper limit.

**Table 5:** Pearson Chi-Square analysis for risk factors associated with leptospirosis.

Factor	Positivity Rate (%)	Pearson Chi-Square	*p*-Value	OR (95% CI)
Outdoor/Indoor	60.8	0.49	0.48	1.40 (0.55–3.59)
Contact with dogs	61.9	0.06	0.80	1.12 (0.46–2.77)
Outdoor food bowl	52.8	2.88	0.09	2.20 (0.88–5.52)
Rodent hunting	64.3	0.03	0.86	1.09 (0.42–2.83)
Bulk food	72	2.64	0.10	2.31 (0.82–6.85)

Note: OR is odds ratio value; 95% CI is 95% confidence interval of lower limit and upper limit.

**Table 6:** Frequency of owner exposure behaviors in dogs positive for *Leptospira* spp. included in this study.

Variable	Yes	%
Kiss on the dog’s face	39	76.5
Kiss on the dog’s mouth	26	51.0
Dog licking the owner’s face	35	68.6
Sleeps in the owner’s bed	13	25.5

## Data Availability

The authors declare that any data not available within the article will be available upon request.

## References

[1] Alberto-Orlando S., Calderon J.L., Leon-Sosa A., Patiño L., Zambrano-Alvarado M.N., Pasquel-Villa L.D., Rugel-Gonzalez D.O., Flores D., Mera M.D., Valencia P. (2022). SARS-CoV-2 transmission from infected owner to household dogs and cats is associated with food sharing. Int. J. Infect. Dis..

[2] Azócar-Aedo L., Monti G. (2015). Meta-Analyses of Factors Associated with Leptospirosis in Domestic Dogs. Zoonoses Public Health.

[3] Bouvet J., Lemaitre L., Cariou C., Scotto M., Blain C., Oberli F., Cupillard L., Guigal P.M. (2020). A canine vaccine against *Leptospira* serovars Icterohaemorrhagiae, Canicola and Grippotyphosa provides cross protection against *Leptospira* serovar Copenhageni. Vet. Immunol. Immunopathol..

[4] Bradley E.A., Lockaby G. (2023). Leptospirosis and the Environment: A Review and Future Directions. Pathogens.

[5] Calvopiña M., Romero-Alvarez D., Vasconez E., Valverde-Muñoz G., Trueba G., Garcia-Bereguiain M.A., Orlando S.A. (2023). Leptospirosis in Ecuador: Current Status and Future Prospects. Trop. Med. Infect. Dis..

[6] Calvopiña M., Vásconez E., Coral-Almeida M., Romero-Alvarez D., Garcia-Bereguiain M.A., Orlando A. (2022). Leptospirosis: Morbidity, mortality, and spatial distribution of hospitalized cases in Ecuador. A nationwide study 2000–2020. PLoS Neglected Trop. Dis..

[7] Carranza Zamora A.J., Chang Fonseca D., Gutierrez López Y. (2020). Leptospirosis y enfermedad de Weil. Rev. Medica Sinerg..

[8] Davignon G., Cagliero J., Guentas L., Bierque E., Genthon P., Gunkel-Grillon P., Juillot F., Kainiu M., Laporte-Magoni C., Picardeau M. (2023). Leptospirosis: Toward a better understanding of the environmental lifestyle of *Leptospira*. Front. Water.

[9] Di Azevedo M.I.N., Santanna R., Carvalho-Costa F.A., Lilenbaum W. (2022). The same strain leading to different clinical outcomes: The enigma behind the canine leptospirosis. Microb. Pathog..

[10] Fávero J.F., de Araújo H.L., Lilenbaum W., Machado G., Tonin A.A., Baldissera M.D., Stefani L.M., da Silva A.S. (2017). Bovine leptospirosis: Prevalence, associated risk factors for infection and their cause-effect relation. Microb. Pathog..

[11] Genevieve A.-F., Laetitia T. (2018). MAT cross-reactions or vaccine cross-protection: Retrospective study of 863 leptospirosis canine cases. Heliyon.

[12] Gizamba J.M., Mugisha L. (2023). Leptospirosis in humans and selected animals in Sub-Saharan Africa, 2014–2022: A systematic review and meta-analysis. BMC Infect. Dis..

[13] Hernández-Ramírez C., Gaxiola-Camacho S., Enriquéz-Verdugo I., Rivas-Llamas R., Osuna-Ramírez I. (2020). Serovariedades de *Leptospira* y riesgos de contagio en humanos y perros de la ciudad de Culiacán, Sinaloa, México. Abanico Vet..

[14] Hilbe M., Posthaus H., Paternoster G., Schuller S., Imlau M., Jahns H. (2024). Exudative glomerulonephritis associated with acute leptospirosis in dogs. Vet. Pathol..

[15] Sykes J.E., Francey T., Schuller S., Stoddard R.A., Cowgill L.D., Moore G.E. (2023). Updated ACVIM consensus statement on leptospirosis in dogs. J. Vet. Intern. Med..

[16] Klaasen H.L.B.M., van der Veen M., Sutton D., Molkenboer M.J.C.H. (2014). A new tetravalent canine leptospirosis vaccine provides at least 12 months immunity against infection. Vet. Immunol. Immunopathol..

[17] Orlando S.A., Mora-Jaramillo N., León Sosa A., Rivera A., Calderon J., Guizado Herrera D., Zevallos J.C., Paredes-Núñez D., Rodriguez-Pazmiño A.S., Carvajal E. (2024). Leptospirosis outbreak in Ecuador in 2023: A pilot study for surveillance from a One Health perspective. One Health.

[18] Knöpfler S., Mayer-Scholl A., Luge E., Klopfleisch R., Gruber A., Nöckler K., Kohn B. (2017). Evaluation of clinical, laboratory, imaging findings and outcome in 99 dogs with leptospirosis. J. Small Anim. Pract..

[19] Lippi I., Puccinelli C., Perondi F., Ceccherini G., Pierini A., Marchetti V., Citi S. (2021). Predictors of fatal pulmonary haemorrhage in dogs affected by leptospirosis approaching haemodialysis. Vet. Sci..

[20] Marami L.M., Gebremedhin E.Z., Sarba E.J., Tola G.K., Endalew S.S., Melkamsew Tesfaye A., di Marco Lo Presti V., Vitale M. (2021). Seroprevalence and Associated Risk Factors of Canine *Leptospira* and *Brucella* Species Infection in West Shewa Zone, Central Ethiopia. Vet. Med. Res. Rep..

[21] Martin E., Heseltine J., Creevy K. (2022). The Evaluation of the Diagnostic Value of a PCR Assay When Compared to a Serologic Micro-Agglutination Test for Canine Leptospirosis. Front. Vet. Sci..

[22] Miotto B.A., Guilloux A.G.A., Tozzi B.F., Moreno L.Z., da Hora A.S., Dias R.A., Heinemann M.B., Moreno A.M., de Souza Filho A.F., Lilenbaum W. (2018). Prospective study of canine leptospirosis in shelter and stray dog populations: Identification of chronic carriers and different *Leptospira* species infecting dogs. PLoS ONE.

[23] Mosquera P., Mejia L., Ortiz G., Pazmino G., Pearson T., Barragán V., Trueba G. (2024). Mixed *Leptospira* infections in domestic animals from a rural community with high leptospirosis endemicity. PLoS ONE.

[24] Novak A., Hindriks E., Hoek A., Veraart C., Broens E.M., Ludwig I., Rutten V., Sloots A., Broere F. (2023). Cellular and humoral immune responsiveness to inactivated *Leptospira interrogans* in dogs vaccinated with a tetravalent *Leptospira* vaccine. Vaccine.

[25] Ordoñez-Álvarez L.Y., Hernández-Bravo R.D.B., Parra-Rodríguez K., Cándano-Acosta A.M., Labrador-Alemán R. (2023). Caracterización clínico epidemiológica de pacientes con leptospirosis humana sospechada Clinical Epidemiological Characterization of Patients with Suspected Human Leptospirosis Artículo Original. Rev. Cienc. Médicas.

[26] Organización Panamericana de la Salud (OPS) (2022). Leptospirosis. Organización Mundial de la Salud. https://www.paho.org/es/temas/leptospirosis.

[27] Orlando S.A., Mora-Jaramillo N., León-Sosa A., Jiménez Valenzuela F., Calderon J., Rivera A., Matamba E., Sanchez E., Macias G., Martinez G. (2025). High prevalence and diversity of *Leptospira* pathogenic serogroups in pigs, cows and free roaming dogs from undeserved rural communities in the coastal region of Ecuador. One Health.

[28] Orlando S.A., Perez A., Sanchez E., de la Cruz C., Rugel O., Garcia-Bereguiain M.A. (2020). High seroprevalence of anti-*Leptospira* spp. antibodies in domestic and wild mammals from a mixed use rescue center in Ecuador: Lessons for “One Health” based conservation strategies. One Health.

[29] Orlando S.A., Sanchez E., Mora-Jaramillo N., Jiménez Valenzuela F., León-Sosa A., Rivera A., Matamba E., Macias G., Martinez G., Piña A. (2025). High prevalence and diversity of *Leptospira* pathogenic serovars in synanthropic fauna from Guayaquil city in Ecuador. Acta Trop..

[30] Pereira M., Valério-Bolas A., Saraiva-Marques C., Alexandre-Pires G., da Fonseca I.P., Santos-Gomes G. (2019). Development of dog immune system: From in uterus to elderly. Vet. Sci..

[31] Polo N., Machado G., Rodrigues R., Hamrick P.N., Munoz-Zanzi C., Pereira M.M., Bercini M., Timm L.N., Schneider M.C. (2019). A one health approach to investigating *Leptospira* serogroups and their spatial distributions among humans and animals in Rio Grande do Sul, Brazil, 2013–2015. Trop. Med. Infect. Dis..

[32] Rahman M.S.A., Khor K.H., Khairani-Bejo S., Lau S.F., Mazlan M., Roslan M.A. (2021). Risk and predictive factors of leptospirosis in dogs diagnosed with kidney and/or liver disease in Selangor, Malaysia. Animals.

[33] Raj J., Campbell R., Tappin S. (2021). Clinical findings in dogs diagnosed with leptospirosis in England. Vet. Rec..

[34] Rajapakse S., Fernando N., Dreyfus A., Smith C., Rodrigo C. (2025). Leptospirosis. Nat. Rev. Dis. Primers.

[35] Ricardo T., Previtali M.A., Signorini M. (2020). Meta-analysis of risk factors for canine leptospirosis. Prev. Vet. Med..

[36] Roulaux P.E.M., van Herwijnen I.R., Beerda B. (2023). Desexing dogs as a means of decreasing the generally regarded sexually dimorphic behaviors of urine marking, mounting, and roaming. J. Vet. Behav..

